# X-Linked retinoschisis associated to a novel intragenic microdeletion: case report

**DOI:** 10.1186/s12881-016-0270-x

**Published:** 2016-01-20

**Authors:** Clara Vazquez-Alfageme, Roberto Reinoso, Alberto Acedo, Rosa M. Coco

**Affiliations:** Instituto de Oftalmobiología Aplicada, Universidad de Valladolid, Valladolid, Spain; NIHR Moorfields Biomedical Research Centre at Moorfields Eye Hospital NHS Foundation Trust and UCL Institute of Ophthalmology, London, UK; Networking Research Center on Bioengineering, Biomaterials and Nanomedicine (CIBER-BBN), Zaragoza, Spain; AC-Gen Reading Life, Valladolid, Spain; Moorfields Eye Hospital, 162 City Rd, London, EC1V 2PD UK

**Keywords:** X-linked retinoschisis, Juvenile retinoschisis, Retinal Degeneration, XLRS, RS1

## Abstract

**Background:**

X-linked retinoschisis is a recessively inherited retinal degeneration. Clinical diagnosis can be challenging due to the highly variable phenotypic presentation. Also, clinical diagnostic tests may be normal at early stages of this condition. Therefore, genetic diagnosis has become a priceless tool in the management of this disease.

**Case presentation:**

We present a case of a 17-year-old caucasian male with foveal and peripheral schisis, along with Mizuo-Nakamura phenomenon. RS1 sequencing led to the discovery of an in-frame deletion not previously described in the literature.

**Conclusions:**

Genetic deletions causative of X-linked retinoschisis are quite rare, since more than 80 % are caused by misssense mutations. In this particular case, its pathological effect comes from affecting a key element of the retinoschisin, the discoidin domain.

## Background

X-linked juvenile retinoschisis (XLRS; OMIM 312700; Online Mendelian Inheritance in Man; http://omim.org/entry/312700 provided in the public domain by the National Center for Biotechnology Information, Bethesda, MD) is an early onset retinal degeneration, characterized by splitting within inner retinal layers. Whereas foveal involvement is present in all affected patients, peripheral schisis takes place in less than 50 % of patients. Electrophysiological testing is key to diagnosis. Full field scotopic electroretinogram (ERG) typically shows preserved a-wave with reduced amplitude of the b-wave indicating an inner retinal dysfunction.

Disease is caused by mutations in a single gene, *retinoschisin 1* (*RS1*; NCBI reference sequence NM 000330), which has been mapped to Xp22. The *RS1* gene contains six exons (NCBI reference sequence: NP 000321.1), and encodes a 224-amino acid–soluble secretory protein, retinoschisin, which is secreted as an homo-octameric complex from photoreceptor and bipolar cells [1–3]. The main structural feature of this protein is the presence of a discoidin-domain, also present is a wide variety of proteins involved in cellular adhesion [4].

Clinical hallmarks of this disease include: mild to severe reduced visual acuity, spoke-wheel pattern in the macula with cystic changes revealed in SD-OCT and, in some cases, peripheral schisis or Mizou-Nakamura phenomemon [5]. Retinal detachment (RD) or vitreous hemorrhage may occur as complications of this condition. XLRS exhibits variable expressivity, even among affected individuals in the same family.

To date 196 different mutations in the RS1 gene are known to cause XLRS (Leiden Open Variation Database, LOVD, version 2.0, Build 35; http://grenada.lumc.nl/LOVD2/eye/home.php?select_db=RS1). More than 80 % are nucleotide substitutions, missense mutations being the predominant type. According to The Retinoschisis Consortium [6] small deletions are not frequent (12 %), have a frameshift effect and are expected to produce truncated proteins with no functional significance. Here, we describe a novel *RS1* mutation in a Spanish family and report genotype findings associated with clinical presentation.

## Case presentation

**Fig. 1:**
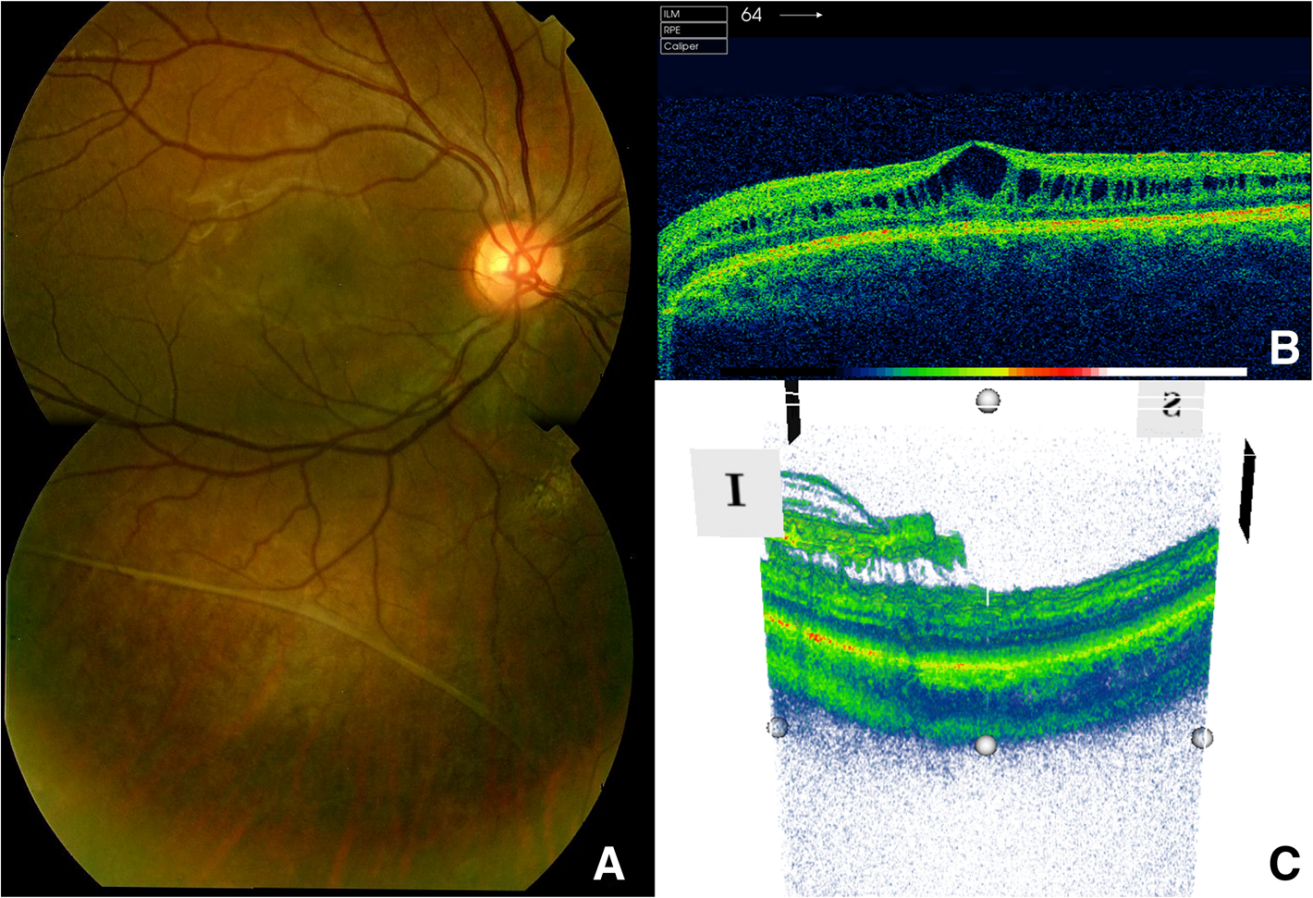
Fundus examination. **a** Peripheral schisis with a large hole can be noticed in the retinography of the right eye. **b** OCT B-scan across the fovea. Intraretinal cysts can be observed along the inner nuclear layer. **c** Three-dimensional reconstruction across the hole in the peripheral schisis of the inferior retina. The inner retinal layers are missed in the right side of the image, while in the left side are still present but detached from the outer retina

17-year-old caucasian male, referred to retina specialists due to progressive bilateral vision loss. There was no past ocular history and his general health was unremarkable. Family history included Age-Related Macular Degeneration (AMD), retinal detachment (RD), glaucoma and high myopia. Best corrected visual acuity (BCVA) was 20/32 in both eyes. Slit lamp examination was unremarkable, but fundus examination revealed bilateral spoke-wheel pattern in the macular area and peripheral schisis with large holes in the inferotemporal quadrant of both eyes. Mizuo-Nakamura phenomenon was also observed in the peripheral retina (Fig. 1).

Spectral Domain Optic Coherence Tomography (SD OCT, Topcon 3D-2000) showed pronounced inner retinal cysts in the foveal area and extended up to the vascular arcades. A negative ERG (Ganzfeld Optoelectronic Stimulator) confirmed the diagnosis of XLRS.

Genetic sequencing analysis revealed a 33-base-pair (bp) deletion (c.467_499GGACCGATGAGCGCCTGAACTGGATTTACTACA) in Exon 5 of the *RS1* gene that results in a non-functional protein consisting in a 11 amino acid deletion (RTDERLNWIYY – p.R156_Y166del). This in-frame deletion mutation has not been previously described and thus, it is not listed in the Retinoschisis Consortium database that is maintained by Leiden University (http://www.dmd.nl/rs/index.html).

**Fig. 2:**
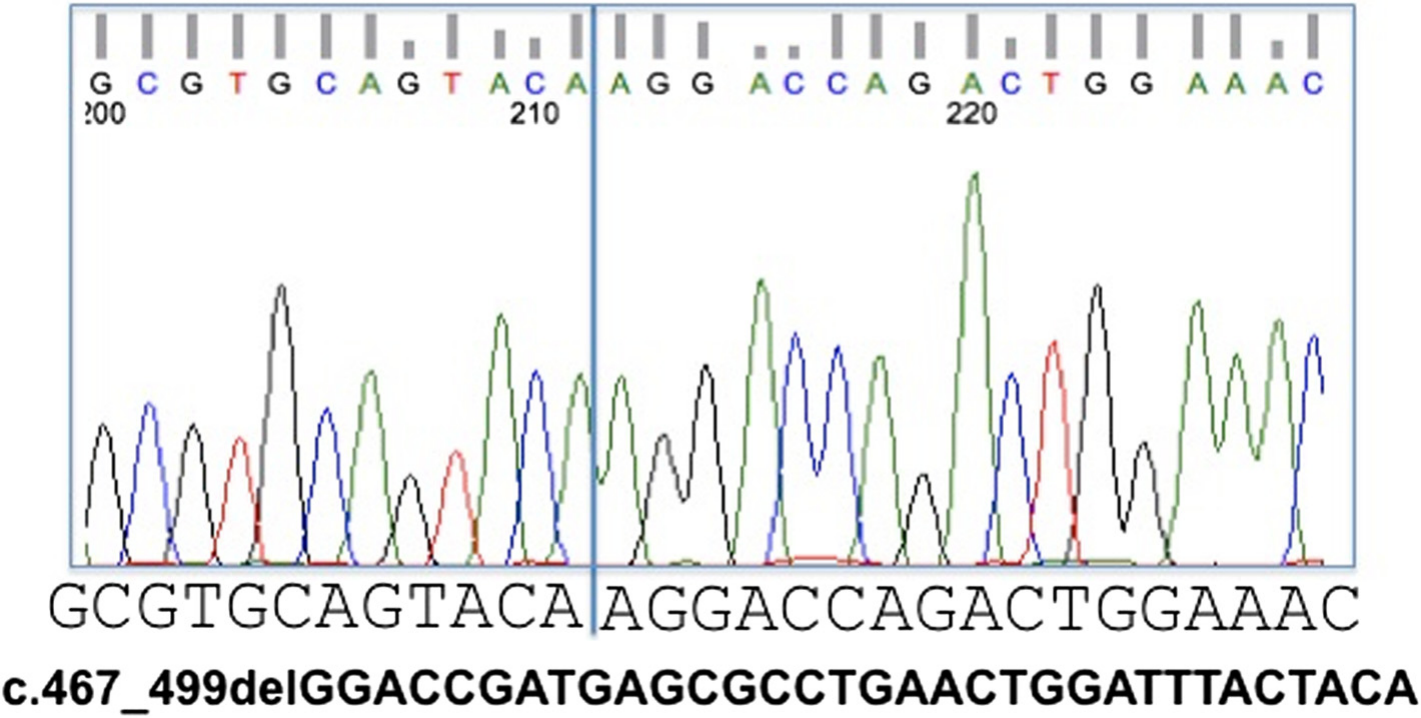
Partial electropherogram of exon 5 of *RS1*. Electropherogram of the proband showing a c.467_499 in-frame deletion mutation of 33 nucleotides leading to 11 amino acid deletion (RTDERLNWIYY – p.R156_Y166del)

**Fig. 3:**
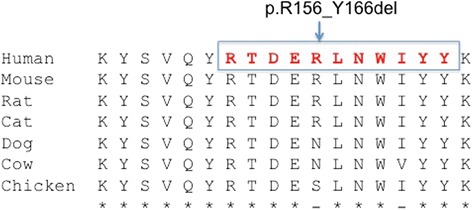
Alignment of RS1 orthologs. It is shown how the deleted sequence is conserved in several mammals, to predict functional importance

Examination of the pathogenicity of c.467_499del33 with PROVEAN (Protein Variation Effect Analyzer) algorithm predicted a deleterious change with a score of −36.77 (Fig. 2 and 3).

The pathological effect of this in-frame deletion may come from the alteration of the core discoidin domain of the RS1. This core is organized as a compact β sandwich containing 5-stranded antiparallel β-sheet (β1, β2, β7, β4, β5) packed against 3-stranded antiparallel β-sheet (β6, β3, β8) [1]. At the end of this β-sheets, spikes or loops are formed, serving as the recognition site for interaction of the discoidin domain with its ligand. The deletion described above affects the final part of the β4-sheet (K150_R156). This change can potentially dislocate the hydrogen bonding between β-sheets essential for proper protein folding, resulting in protein misfolding and endoplasmic reticulum (ER) retention. Therefore, the phenotype would arise from the loss in functional protein.

It has been previously reported that disease-causing mutations in the core barrel structure cause protein misfolding and ER retention, even when they do not involve cysteine residues responsible for the disulphide bridge formation inside the protein.

## Conclusions

The patient reported here showed an in-frame microdeletion not previously described linked to XLRS. This case emphasizes the critical functional significance of the discoidin domain of the XLRS1 protein.

### Consent

Written informed consent was obtained from the patient for publication of this Case report and any accompanying images. A copy of the written consent is available for review by the Editor of this journal.
